# Amelogenin Peptide-Chitosan Hydrogel for Biomimetic Enamel Regrowth

**DOI:** 10.3389/fdmed.2021.697544

**Published:** 2021-06-16

**Authors:** Kaushik Mukherjee, Amrita Chakraborty, Garima Sandhu, Sohaib Naim, E. Bauza Nowotny, Janet Moradian-Oldak

**Affiliations:** 1 Center for Craniofacial Molecular Biology, Herman Ostrow School of Dentistry, University of Southern California, Los Angeles, CA, United States; 2 Department of Biomedical Engineering, Whiting School of Engineering, Johns Hopkins, Baltimore, MD, United States

**Keywords:** hydroxyapatite, remineralization, caries, QLF, enamel, bioactive peptide

## Abstract

We designed synthetic peptides that have demonstrated an effective remineralization potential to restore incipient enamel decay. In order to develop a clinically viable approach we incorporated the amelogenin-derived peptides P26 and P32 into chitosan hydrogel and examined their efficacy in the remineralization of enamel. Peptides in chitosan exhibited increased stability *in vitro* as compared to peptides in solution at room temperature and at 37°C. Tooth models for enamel erosion (sections) and white spot lesions (blocks) were subject to periods of demineralization. Treatment groups were subjected to remineralization in artificial saliva in the presence of P26 and P32 in solution and in chitosan hydrogel (P26-CS and P32-CS). Quantitative light-induced fluorescence (QLF) was employed to analyze mineral density following demineralization and remineralization across all the treatment groups. Scanning electron microscopy and nanoindentation were used to characterize the surface structure and mechanical strength of regrown enamel. Control enamel sections treated in artificial saliva demonstrated randomly distributed, tiny, needle-shaped crystals with a low packing density and porosities displaying mineralization defects. In samples treated with P26-CS or P32-CS a denser coating of organized hydroxyapatite (HAP) crystals was formed covering the entire surfaces of demineralized enamel window. The hardness and modulus of enamel surfaces were increased after treatment with P26-CS and P32-CS with no significant difference in the mechanical properties between the two peptide hydrogels. Analysis of mineral density by QLF showed that in enamel sections P26 peptide alone or P26-CS significantly enhanced the remineralization. In enamel blocks P26 in solution had a better efficacy than P26-CS.

## INTRODUCTION

In dental caries, the localized destruction of susceptible dental hard tissues (enamel, dentin, cementum) begins as initial dissolution of mineral at the sub-clinical stage followed by incrementally progressive destruction visible only as an early lesion, that finally leads to cavitation (1). White spot lesions (WSL) on the other hand are subsurface enamel lesions that develop as a result of gradual enamel demineralization, and are characterized by their opaque, chalky white appearance (2).

Early and successful treatment of mineral loss in enamel lesions, whether they are the result of dental caries, attrition, abrasion, or erosion is a critical step toward prevention of tooth decay and avoidance of invasive dental procedures (3). There are multiple treatment modalities for WSL including professional fluoride application, casein phosphopeptides (4), and infiltrative resins (5, 6). While fluoride application has been traditionally the most common therapy for WSLs, mineralization by high concentrations of fluoride is limited to the enamel surface and prevents movement of ions into the subsurface lesion. CPPs stabilize the calcium and phosphate ions in saliva to form CPP-Amorphous Calcium Phosphate (CPP-ACP) complex thus promoting remineralization. Infiltrative resins involve low-viscosity resin penetration into etched surfaces of the enamel lesion to mechanically stabilize the hydroxyapatite structure. Though these and other commercially available treatment modalities have been effective in partially treating WSLs, their effectiveness is still limited (7). Biomimetic remineralization of enamel and dentin using bioactive peptides have been recently considered as an alternative to the conventional treatment modality such as fluoride and ACP-based products (3, 8-13).

We have used amelogenin containing chitosan hydrogels for ex vivo biomimetic enamel regrowth (14, 15). We demonstrated that recombinant enamel protein amelogenin (rP172) and a leucine-rich amelogenin polypeptide (LRAP) incorporated into chitosan hydrogel captured the calcium and the phosphate from the artificial saliva, guiding them to sequentially deposit oriented HAP layers on the surface of demineralized enamel. The newly formed enamel-like layer had a robust, seamless interface with the natural tooth. Chitosan was used as a medium to deliver the peptides due to its multifold properties such as biocompatibility, biodegradability, and its bactericidal and bacteriostatic properties through accumulation of positive charges on tooth surfaces (16). This led to the design of bioinspired amelogenin peptides P26 and P32 with retained functional domains capable of regenerating tooth microstructures, enamel and dentin. When applied on dental tissues, these peptides demonstrated an effective remineralization potential to restore incipient enamel decay and mineralization defects localized in peripheral dentin (17). Repeated peptide applications led to multiple microscale enamel-like layers, forming an organized aprismatic enamel while imparting significant mechanical properties to treated enamel. Furthermore, application of the P26 on demineralized dentin sections prompted P26 and collagen interactions, thus promoting collagen remineralization and enhancement of the mineral density and mechanical strength of demineralized dentin (18).

Here, we report a systematic comparison in remineralization effectiveness of P26 and P32 peptide in solution vs. the peptides incorporated into chitosan hydrogel (P26-CS and P32-CS) in enamel sections and blocks. *In vitro* assessment of hydrogel stability showed that peptides in chitosan (P26-CS and P32-CS) were more resistant to degradation than the peptides in solution both at room temperature (RT) and at 37°C. We used enamel sections as a model for erosion and enamel blocks as a model for WSL. The artificial lesions were analyzed for mineral density gain by using quantitative light induced fluorescence camera (QLF) (19, 20). To assess the surface topography and strength of the remineralized layers, the repaired enamel sections were analyzed by SEM and nanoindentation, respectively.

## MATERIALS AND METHODS

### Preparation of Sections

Tooth samples were prepared from extracted teeth without any pre-existing caries or white spot lesions (extracted using standard procedures at the Herman Ostrow School of Dentistry of the University of Southern California). The teeth were stored in Phosphate-buffered saline (PBS, pH 7.4) in 0.002% sodium azide and later washed with de-ionized water and sonicated (Branson M1800 Ultrasonic Cleaner) for 20 min.

#### Enamel Sections

Longitudinal tooth sections measuring 1.5 mm (+/−0.2 mm) in thickness were derived from each tooth by sectioning, using low-speed diamond saw (MTI Corporation SYJ160, USA), polished using a fine grid (2,000 grid) sandpaper and sonicated for 5 min. The surface of each sample was coated with acid resistant nail varnish leaving two exposed windows on each section measuring 1 mm × 2 mm. The samples were dried for 2 h after varnish application.

#### Enamel Blocks

Each tooth was divided into 4 enamel blocks and coated with a clear acid-resistant nail varnish leaving an exposed window of 2 mm × 2 mm in the enamel.

### Peptide Preparation (in Solution and in Chitosan Hydrogel)

Five experimental groups were compared, which included control, peptides P26 and P32 in solution and incorporated in chitosan hydrogel (P26-CS and P32-CS). The rationally designed peptides were synthesized commercially at ~95.13% purity by CHEMPEPTIDE Limited (Shanghai, China). The peptides were phosphorylated at serine-16 (Supplementary Figure 1).

Peptide solutions were prepared as described previously (21) at a concentration of 0.2 mg/mL (200 μg of peptide in 960 μL of ultra-pure water with 25 μL CaCl_2_ and 15 μL Na_2_HPO_4_ at pH 6.50). Peptides incorporated in a chitosan hydrogel were prepared at a concentration of 0.2 mg/mL (200 μg of peptide in 960 μL of chitosan with 25 μL CaCl_2_ and 15 μL Na_2_HPO_4_ at final pH 6.50). For enamel blocks, the concentration of P26 solution was increased to 0.5 mg/mL (500 μg of peptide in 960 μL of ultra-pure water with 25 μL CaCl_2_ and 15 μL Na_2_HPO_4_ at pH 6.50).

### Demineralization and Remineralization Cycles for QLF

Artificial demineralized lesions were created by immersing the enamel sections in demineralization buffer (2mM CaCl_2_, 2mM KH_2_PO_4_, 50mM sodium acetate, and 0.05M acetic acid at pH 4.6 at 37<C) for 3 days, and for WSLs the enamel blocks were immersed for 15 days. Post demineralization, the samples were rinsed with de-ionized water, sonicated for 5 min to remove all residual debris and air-dried for 20 s.

Remineralization was achieved in artificial saliva (1.2 mM CaCl_2_·2H_2_O, 50 mM HEPES buffer, 0.72 mM KH_2_PO_4_, 16 mM KCl, 4.5 mM NH_4_Cl, 0.2 mM MgCl2·6H_2_0, and 1 ppm NaF) across the following experimental groups: peptide only, P-CS and control (artificial saliva). For each group, samples were air dried for 20s followed by application of 40μL of peptide treatment. The enamel sections were remineralized for 7 days with peptide applications on day 1 and 3. Enamel blocks were remineralized for 21 days with peptide applications on day 1, 7 and day 14. All the samples were incubated at 37°C in 5 mL of artificial saliva at pH 7.0. The artificial saliva was replenished every 24 h (21).

### Quantitative Light Induced Fluorescence (QLF) System

QLF images were taken under white light (ISO 1,600, aperture 18, shutter speed 1/125s) and under blue light (ISO 1,600, aperture 5.6, shutter speed 1/30s) (22). These settings were kept constant throughout the project. For each QLF image, the region of interest was compared with the surrounding sound enamel by creating a contour that included the lesion and surrounding sound tissue. Once completed, the software automatically generated a value for the circumscribed lesion based on fluorescence loss in comparison with the sound tissue. For the purpose of visual identification of white spot lesions, the white light image was considered. However, for this study, assessment and quantification of mineral density, changes were based on the ΔF values obtained from the blue light images. QLF images were taken at 3 different time points and each reading repeated three times (baseline, post-demineralization, and post-remineralization) for enamel sections (*n* = 7) and enamel blocks (*n* = 12). The demineralized enamel lesions manifested as dark gray areas on the QLF-D images, indicating a decrease in fluorescence radiance and mineral content. Statistical analysis included one-way ANOVA and Tukey’s test for multiple comparisons. Both quantitative and qualitative analyses of the samples were performed. For the quantitative analysis, the parameters considered were as follows:

ΔF: Percentage loss of fluorescence with respect to the fluorescence of sound tooth tissue (mineral density).

ΔΔF: ΔF_(Demin)_ – ΔF_(Baseline)_ or ΔF_(Remin)_ – ΔF_(Demin)_; A negative ΔΔF implies mineral loss and a positive ΔΔF value implies mineral gain.

For the qualitative analysis, a decrease in gray patches in the experimental window represented a decrease in demineralization. Visual comparison of images at baseline, post demineralization and post remineralization was performed to assess for decreased grayness.

### Scanning Electron Microscopy (SEM)

At the end of 3 day *in situ* remineralization cycle (17), field emission SEM (JEOL JSM-7001F, JEOL Ltd., Tokyo, Japan) imaging was used to observe the regrown incipient HAP crystals for structural analysis and comparison. Tooth specimens were sputtered with Au before observation and viewed under an accelerating voltage of 10 kV.

### Mechanical Properties and Nanoindentation

To enhance the mechanical responses, the remineralization cycle was optimized and extended to 7 days. After demineralization, each tooth setting (comprising 3 or 4 teeth sections) was placed into separate cylindrical containers containing remineralization solution (30 ml, pH 7, RT) for 1 week. Experimental groups included healthy enamel, demineralized enamel, control (900 ppm F^−^; 0.2% NaF), P26-CS and P32-CS (*n* = 3 per group).

Every 24 h after the start of the remineralization cycle, 30 μL of NaF solution (900 ppm F^−^; 0.2% NaF) was added on top of each tooth for 10 min per day. The teeth were gently rinsed in distilled water and dried. A fresh 20 μl treatment application (P26-CS and P32-CS) was made to each window, followed by air drying for 15 min at RT and then the samples were returned to the fresh stock of remineralization solution. The remineralization solution and peptide were replaced daily. After 1 week of daily treatment application, the slices were sonicated, rinsed, dried, and stored in distilled water at 4°C for nanoindentation. The hardness and elastic modulus of the peptide-mediated mineralized layers were evaluated by nanoindentation tests. The device was calibrated to examine the mechanical properties of the remineralized HAP layers on teeth slices at the nanoscale. The load placed on the new TB26980 CSM probe was adjusted according to the indentation depth, geometry of the specified location, and the number of indents being made. Specifications detailed for nanoindentation used in CSM mode included: indent depth of 2000 nm and a target constant strain rate of 0.05 s^−1^ on an enamel window area of 2*2 mm. Every tooth slice received 50 indents spaced at 50 μm intervals. One-way ANOVA and Tukey’s tests were applied to identify differences in the hardness and elastic modulus between etched and repaired enamel. Statistical analyses were performed using GraphPad Prism 9.1 (San Diego, CA).

### *In vitro* Peptide Stability

Solutions of amelogenin-derived peptides P26 and P26-CS (0.2mg/mL), P32 and P32-CS (0.4 mg/mL) were prepared as described previously by (14, 17). To assess the stability of the peptides in solution and in chitosan hydrogel, the solutions were left at benchtop or incubated at 37°C and a small volume (100 μl) was collected from each sample at selected time points (0, 1, 7, and 28 days) and diluted in 100 μl of 0.1% TFA. Each collection was subsequently analyzed via reversed-phase HPLC using an analytical C18 column. The amelogenin peptide peaks of P26 and P32 were analytically detected via HPLC and their area were measured to demonstrate stability of the peptides, both in solution (P26 and P32) and in chitosan (P26-CS and P32-CS). Statistical analyses were performed using GraphPad Prism 9.1 (San Diego, CA).

## RESULTS

### Remineralization of Enamel Sections Observed by SEM

**FIGURE 1 ∣:**
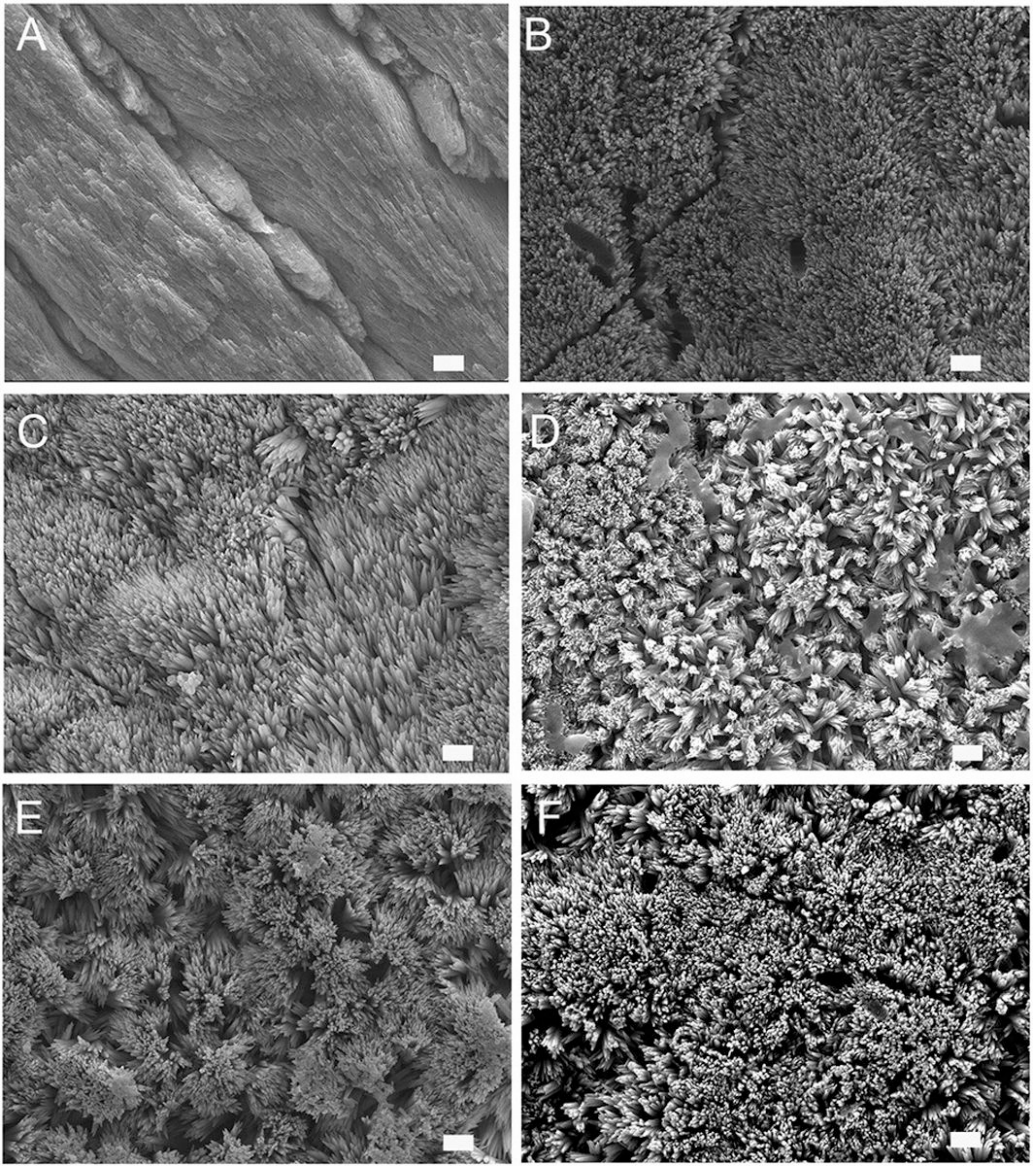
SEM images of demineralized enamel surface showing clear outlines of enamel prisms/rods with remnants of inter-rods **(A)**. HAP crystals grown on demineralized enamel after 3 days of incubation at pH 7.0, 37 °C in artificial saliva (control) **(B)**, with P32 **(C)**, P32-CS **(D)**, P26 **(E)**, P26-CS (F) (scale = 1 um).

Human enamel sections were used as a model for enamel erosion and subjected to a 2 h demineralization cycle at pH 4.6. The enamel slices were eroded and showed a distinct smooth enamel prism pattern with remnants of inter-rods at the prism boundaries (Figure 1A). SEM was used to analyze the alterations in crystal growth, orientation, and morphology on the enamel surfaces post remineralization. The control samples with no treatment were incubated in artificial saliva for 2–7 days. As depicted in Figure 1B, the control samples treated in artificial saliva for 3 days demonstrated randomly distributed, tiny, needle-shaped crystals with a low packing density and porosities displaying mineralization defects. However, samples treated either in P32 or P32-CS and P26 or P26-CS (Figures 1C-F) for 3 days in artificial saliva a denser coating of HAP crystals was formed covering the entire surface of demineralized enamel window. To investigate if the presence of chitosan could alter the structure-function dynamics in the remineralization cycle, enamel slices were treated with P32 only (no chitosan) and incubated for 2–3 days (Figure 1C). The initial mineral layout showed a rapid crystal overgrowth with bundles of needle-like crystals emerging parallel to the underlying prismatic enamel and uniformly coating the acid-etched enamel surfaces (Figures 1C,D). Remnants of chitosan-based hydrogel scaffolds could be seen both on the surfaces and entrapped between the forming crystals, which may have additional roles in their biological and functional responses (Figure 1D). Enamel slices incubated in P26-CS and P26 only depicted a similar pattern of crystal morphology, orientation, and mechanical responses as their counterpart P32-CS and P32 (Figures 1E,F). As the duration of the mineralization cycle progressed in the presence of the peptides (for up to 7 days), there was a noticeable increase in the density, thickness, and attachment of the newly formed crystals at the interface to underlying enamel.

### Efficacy of Peptide-Hydrogel in Improving Hardness and Elastic Modulus of Demineralized Enamel Sections

**FIGURE 2 ∣:**
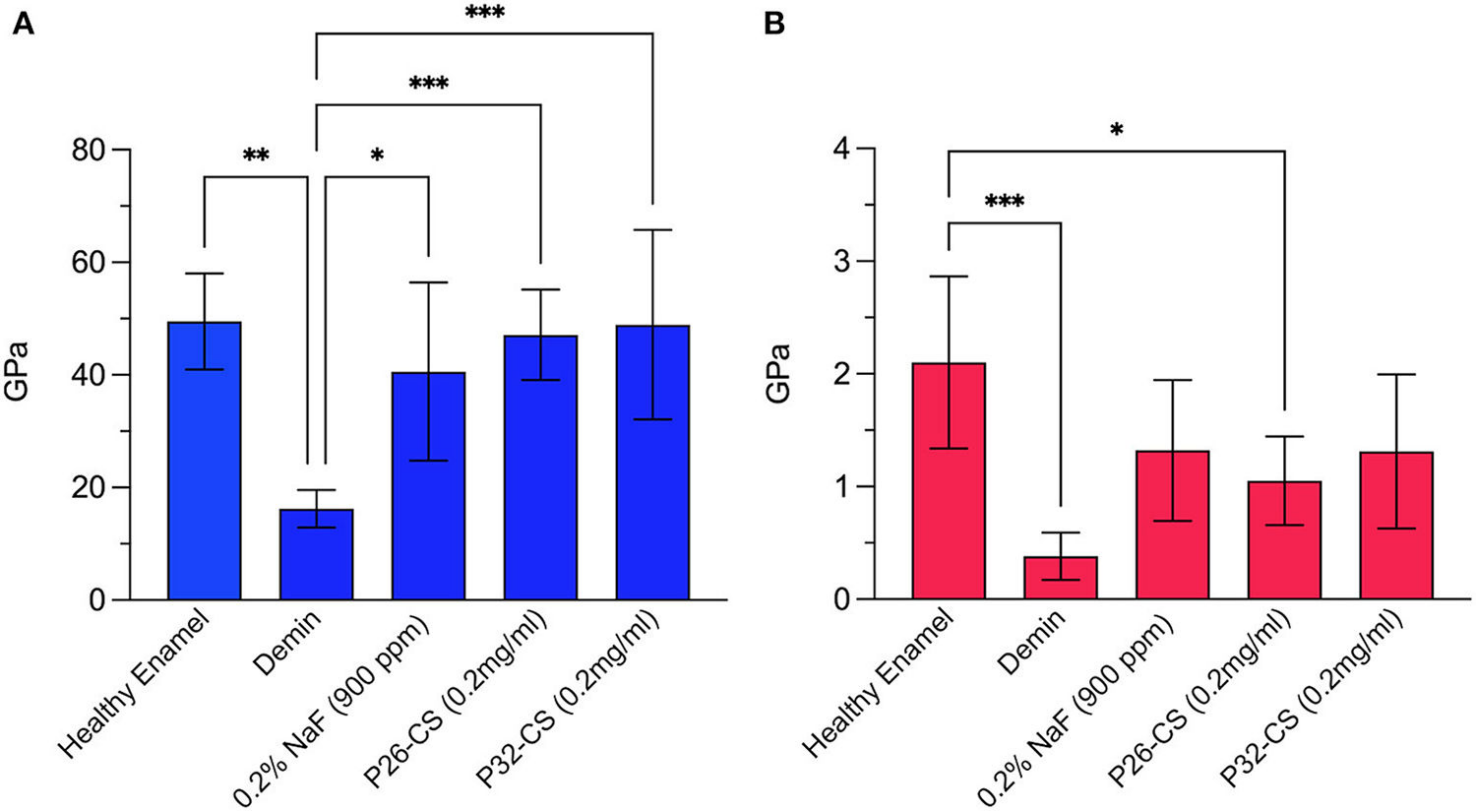
Nanoindentation data showing the modulus **(A)** and hardness **(B)** for healthy enamel, demineralized enamel, and samples treated in control (0.2% NaF), P26-CS (0.2 mg/ml) and P32-CS (0.2 mg/ml) hydrogel for 7 days in artificial saliva. The hydrogel applications were made daily. Demin: demineralization (2 h). The error bars represent standard deviation (*n* = 5–8 per group). Tukey’s multiple comparisons test was applied to identify differences in the hardness and elastic modulus between demineralized and repaired enamel. * ≤ 0.05; ** ≤ 0.01; *** ≤ 0.001.

Demineralization of the enamel sections for 2 h at pH 4.6 resulted in significant erosion and reduction in mechanical strength (Figure 2). After a 7-day mineralization cycle in artificial saliva, regrown enamel crystals in P26-CS demonstrated a 2.9-fold increase in modulus and a 2.6-fold increase in hardness when compared to demineralized enamel (47.12 ± 8GPa; 1.05 ± 0.3GPa). P32-CS treated samples showed a similar pattern with a 3-fold increase in modulus and 1.7-fold increase in hardness (48.85 ± 16.8GPa; 1.3 ± 0.6GPa). There was no statistical difference in the mechanical properties between the peptide treated samples (*p* > *0*.05) indicating similar biomechanical responses. Importantly, the gain in elastic modulus of repaired enamel was significant (*p* < 0.001) up to 96% while the recovery in hardness was approximately 62% when compared to healthy enamel. When compared to the peptide treated samples, the control samples treated in 0.2% NaF showed a smaller increase in modulus and a similar increase in hardness (40.57 ± 15.8GPa; 1.3 ± 0.6GPa).

### Evaluation of Mineral Density of Remineralized Enamel Sections and Blocks by QLF

**FIGURE 3 ∣:**
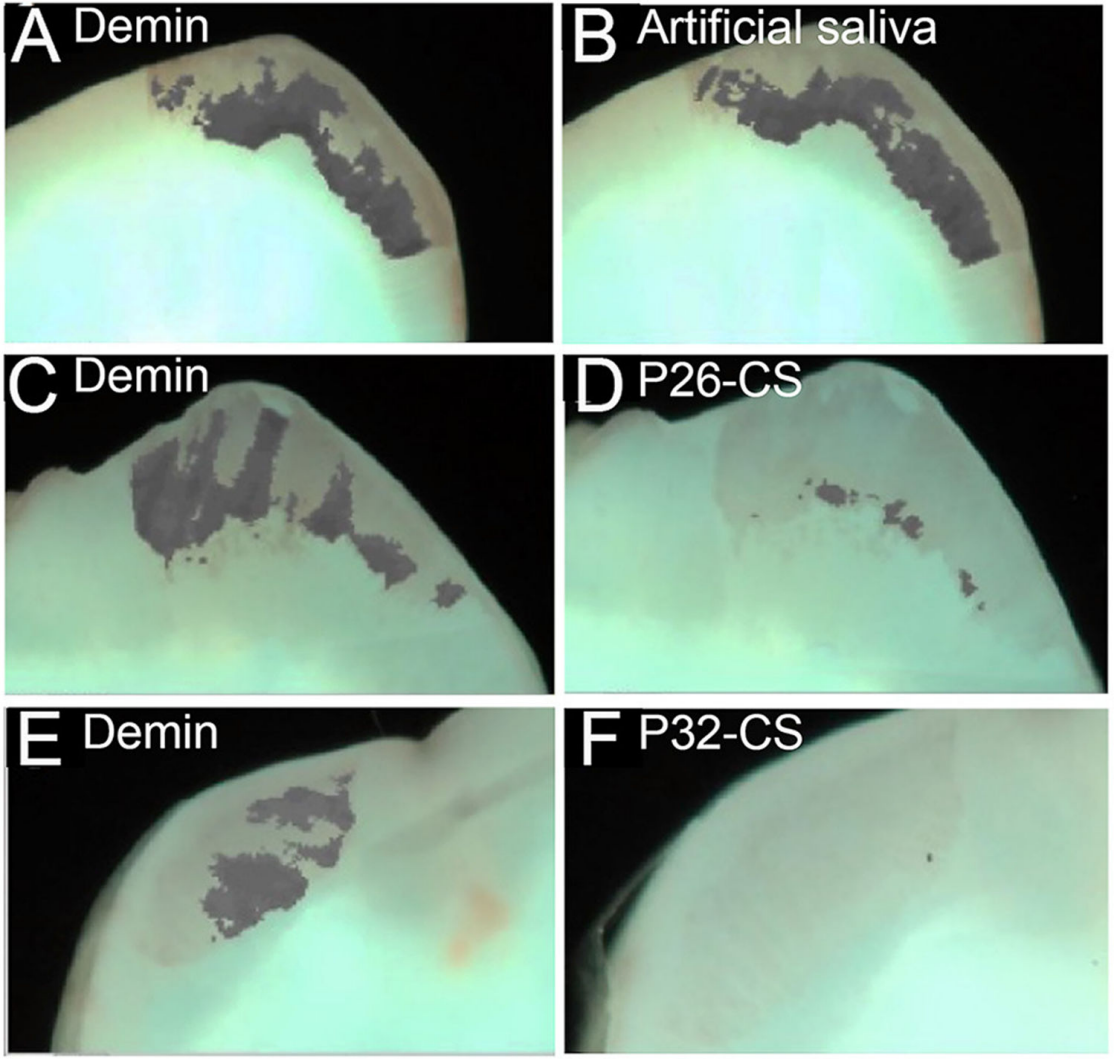
Representative white-light images of the visual comparisons made between the three experimental groups tested. Demineralized samples **(A, C, E)** and remineralized samples **(B, D, F)**. The images clearly show a minimal visual decrease in the gray areas in the control remineralized in artificial saliva only **(B)**, whereas there is visual decrease in the gray patches (mineral gain) of the enamel sections post remineralization with P26-CS **(D)** and P32-CS **(F)**.

Figures 3A-F depicts demineralization and remineralization images of tooth sections taken by white-light QLF imaging showing areas of demineralized windows as identifiable gray spots around the cuspal tips. The quantity Δ*F*, defined as the percentage change in fluorescence radiance in each image point, represented the mean mineral loss of the selected area on the sectioned tooth slices. The baseline Δ*F* for sound tooth enamel ranged between 0 to −6.3, with 86% of the samples reading a 0 value while Δ*F* after demineralization was −32 ± 7.4 (Figures 3A,C,E). Post remineralization, the control samples had no impact in mineralization recovery (Figure 3B). However, as demonstrated by a substantial reduction in Δ*F* (d,f), P26-CS and P32-CS were equally effective in their remineralization potential supporting our previous studies. The conspicuous gray spots were completely resolved indicating gain in mineral density. Hence, for further statistical analysis, we focused on the smaller of the two peptides, namely P26.

**FIGURE 4 ∣:**
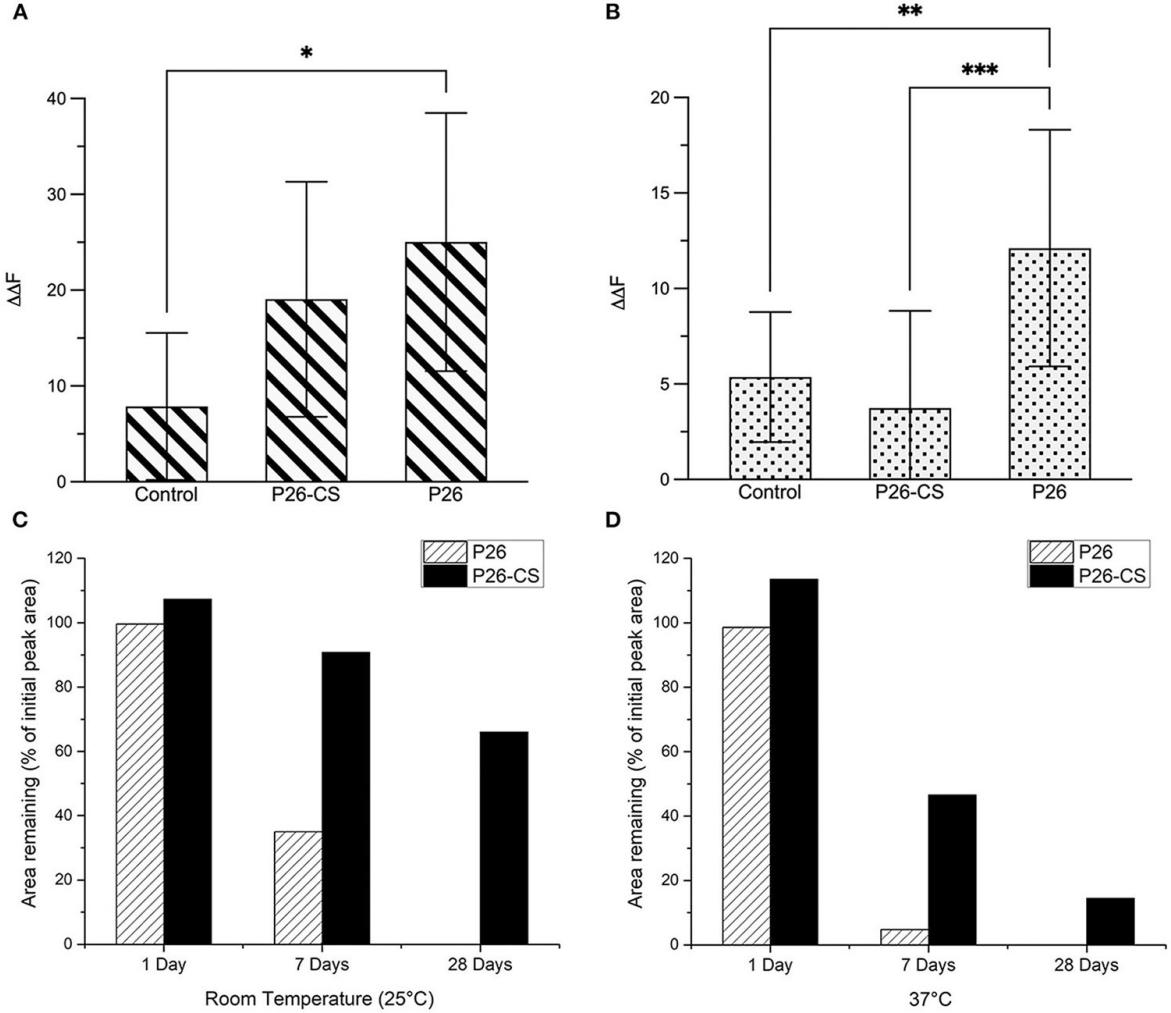
Evaluation of Remineralized Enamel Mineral Density and stability of amelogenin peptides. Comparative quantitative analysis of ΔΔF_remin_ between the three experimental groups before and after remineralization: control, P26-CS and P26. In enamel sections **(A)** there was no significant difference between the P26 solution and P26-CS treatment group (*p* > 0.05). In enamel blocks **(B)** there was a highly significant difference between P26 solution and control group (*p* < 0.007). Percentage of peptide remaining after 1, 7, and 28 days in P26 and P26-CS solutions (*n* = 3 per group) incubated at room temperature **(C)** and 37°C **(D)**. While P26 and P26-CS were comparably stable after 1 day, P26-CS is remarkably more stable after 7 and 28 days at RT and 37°C, as indicated by a greater % area. * ≤ 0.05; ** ≤ 0.01; *** ≤ 0.001.

Figure 4A represents the comparative quantitative analysis of gain in mineral density (ΔΔF) between the 3 experimental groups in enamel sections, control, P26-CS and P26 only. Changes in fluorescence luminance (ΔΔFremin) resulting from enamel demineralization and remineralization can be used as an analogous measure of mineral density. The highest remineralization was observed in the P26 solution group (ΔΔF = +25 ± 13.4) followed by P26-CS (ΔΔF = +19±12.2) and being the lowest in the control (ΔΔF = +7.8±7.6). One-way ANOVA indicated that ΔΔF across the three treatment groups was significant (*p* = 0.035). Additionally, no significant difference was present between P26 and P26-CS (*p* > 0.5). These results suggest that the P26 peptide alone or incorporated into chitosan can significantly enhance the remineralization of enamel under our experimental conditions. Additionally, the model for WSL with human enamel blocks showed a significant gain in mineral density after treatment with P26 (*p* = 0.0066; Figure 4B).

### Stability of Amelogenin Peptides P26 and P32 in Solution and in Chitosan

We examined the stability of amelogenin peptides in solution and in chitosan at room temperature and at 37°C using HPLC. Results show that P26 and P26-CS were substantially stable after a 1-day incubation at RT and 37°C (Figures 4C,D). After 7 days, P26-CS demonstrated considerable stability at RT and 37 °C with 86% and 41.6% of initial peak area remaining, respectively (Figures 4C,D). In contrast, P26 was less stable than P26-CS with 32% area remaining at RT and only about 5% at 37 °C (Figures 4C,D). P26-CS remained fairly stable at RT and 37° C after 28 days while P26 was characterized by the absence of the amelogenin peak and appearance of new peaks (Supplementary Figure 2).

Statistically significant differences were observed for the effect of time and treatment group (P26 or P26-CS) on the observed peak area at RT (*p* < 0.0001) and 37°C (*p* < 0.0001) by two-way ANOVA. Additionally, the data show that, after Bonferroni correction for multiple testing, the mean peak area was significantly different between P26 and P26-CS at 0 days (adjusted *p* < 0.0001), 1 day (adjusted *p* = 0.0165), and 7 days (adjusted *p* < 0.0001) at 37 °C, but no other pairwise group comparison was statistically significant (Supplementary Figure 3). Importantly, P26-CS and P32-CS (not shown) showed a smaller decrease in peak area than their respective peptides after 1, 7, and 28 days, thus suggesting greater stability of the peptides in chitosan than in solution (Figures 4C,D).

## DISCUSSION

In order to develop a clinically viable approach for biomimetic enamel remineralization we incorporated our previously designed amelogenin-derived peptides into chitosan hydrogel (P26-CS and P32-CS) and examined their efficacy in remineralization of enamel sections and enamel blocks. We have previously reported that the synthetic peptides P26 and P32 are intrinsically disordered and formed a characteristic nanostructured scaffold reminiscent of “nanospheres” similar to those formed by the full-length amelogenin (17).

Application of chitosan on enamel surfaces can entrap calcium ions from the artificial saliva and coupled with the self-assembled peptides may serve as an effective prototype to deliver mineral ions in incipient caries. Functional role of chitosan gel includes increased adhesion of repaired enamel, providing a seamless interface that may potentially improve the durability of restorations and curb the formation of secondary caries (14). Chitosan has antibacterial properties against specific cariesforming bacteria like streptococci and lactobacilli, that may render the newly-formed enamel crystals to be more resistant to enamel demineralization by bacterial activity (14, 16, 23). Additionally, chitosan is less toxic to mammalian cells which offers additional advantages *in vivo*. Here, we used HPLC to analyze P26-CS and P32-CS hydrogel stability following incubations at 1, 7, and 28 days to show that chitosan enhances the stability of P26 and P32 peptides while greatly reducing degradation. Such protective biological and functional actions of chitosan ensure prolonged peptide viability in the oral cavity that can help us in further optimizing the minimum effective concentration and frequency of application to combat dental caries.

The P26 and P32 peptides have been designed based on the active domains of enamel protein amelogenin including the N-terminal self-assembly domain, apatite binding domain and the phosphorylated serine (Supplementary Figure 1). The potential of the P26 peptide in repairing enamel and dentin has been reported in our most recent studies which show an organized growth of enamel-like crystals on the surface of demineralized enamel with an improved integration at the regrowth interface (17). The regenerated apatite had a composition (i.e., Ca/P ratio) comparable to that of native enamel (17). Examining the biomechanical properties, a 1.7-fold increase in elastic modulus and a 1.8-fold increase in the hardness of the enamel sample in comparison with the demineralized enamel were observed. In the present study the mechanical properties were evaluated to assess the strength of the hydrogel-treated remineralized enamel sections. We particularly demonstrate that incorporating the peptides into chitosan did not affect the potential of the peptide to enhance mechanical properties. We have previously shown that chitosan alone does not contribute toward a significant improvement in the biomechanical properties of treated enamel surfaces (15). There was no statistical difference in the mechanical properties between the peptide only treated samples and chitosan containing peptide samples (*n* = 3; *p* >> 0.05) indicating similar biomechanical responses. Additionally, no statistically significant differences were observed between P26 and P32. Therefore, for the analysis of mineral density using QLF we focused on the ability of the shorter peptide P26 and P26-CS to remineralize demineralized enamel sections and blocks. The enamel sections were used as a model for enamel erosion while enamel blocks were used to mimic the clinical problem of white spot lesions.

QLF system serves as an optimal tool to study changes within enamel lesions overtime. Previous studies have demonstrated QLF to have high sensitivity for quantification of mineral loss with high correlation to changes in mineral content. The images once stored in the QLF system can be assessed at any time. This proves extremely beneficial for future clinical trials when progression of lesions and their remineralization can be analyzed over time. It has been noted that the white spot lesions do not exhibit any autofluorescence (24). This explains the highly negative ΔF values recorded by QLF for demineralized enamel blocks reported here, which necessitates the use of supplementary techniques to evaluate crystal growth and density on remineralized surfaces. In enamel blocks, remineralization could be a product of subsurface and superficial remineralization and needs additional studies to assess peptide penetration.

As observed by SEM images of initial crystal growth on enamel sections, artificial saliva alone initiated early HAP crystal formation, likely attributable to the variation in charges between the free mineral ions in artificial saliva (calcium and phosphate ions) and the negative-positive charged domains on enamel surfaces. However, without a mediator (such as amelogenin-derived peptides), the mineralization process can be haphazard. This observation was validated through the samples treated in P32-CS or P26-CS for 3 days in artificial saliva wherein a denser coating of organized HAP crystals was formed covering the entire surface of demineralized enamel window. Amongst the three treatment groups used in the analysis of mineral density, P26 in solution was most effective in improving mineral density in enamel blocks. As mentioned previously, P26 aids in the regeneration of apatite like crystals on the surface of demineralized samples. This regeneration potential of P26 was also observed in the enamel erosion model (enamel sections) and WSL model (enamel blocks). Therefore, it is evident that P26 in solution form has better penetration potential in comparison to P26 in chitosan hydrogel. P26-CS was selected as the final prototype for clinical use due to the additional benefits of incorporating chitosan as a delivery agent. In order to improve the efficacy of P26-CS for clinical studies, mild etching will be recommended prior to application of the hydrogel to enhance peptide penetrability into deeper lesions. Further research needs to be directed toward the penetration potential of P26 in subsurface enamel lesions, in both solution and chitosan hydrogel.

## CONCLUSION

We introduced a peptide-based hydrogel to treat incipient enamel caries. This prototype combines amelogenin peptide-mediated enamel remineralization principle with the antimicrobial properties of chitosan that enhances peptide stability. Rationally designed amelogenin peptides act as effective modulators of apatite nucleation and crystal growth processes, giving rise to oriented enamel-like HAP crystals with increased biomechanical properties on the surfaces of demineralized enamel. QLF was employed as an effective clinical assessment tool to draw a comparative analysis of enamel de and remineralization events after the application of the peptide and peptide-chitosan hydrogel. From a clinical standpoint, this biomimetic translational approach can be optimized for the development of novel complex biomaterials.

## Supplementary Material

Supplementary material

## Data Availability

The datasets generated for this study can be found in online repositories. The names of the repository/repositories and accession numbers can be found in the article/Supplementary Material.

## Abbreviations:

HPLC: High Performance Liquid Chromatography
P26: 26 amino acid Amelogenin-derived peptide
P32: 32 amino acid Amelogenin-derived peptide
P26-CS: P26 in chitosan
P32-CS: P32 in chitosan
QLF: Quantitative light-induced fluorescence camera
LRAP: Leucine-Rich Amelogenin Polypeptide
WSL: White Spot Lesions
