# Supplementary material for: Amelogenin Peptide-Chitosan Hydrogel for Biomimetic Enamel Regrowth
Source: Front Dent Med. Author manuscript; Available in PMC 2023 Oct 27. (PMC10611442; doi:10.3389/fdmed.2021.697544)
Supplement: Supplementary material [file NIHMS1890649-supplement-Supplementary_material.docx]

**Amelogenin peptide-chitosan hydrogel for biomimetic enamel regrowth**

**(Kaushik Mukherjee, Amrita Chakraborty Garima Sandhu, Sohaib Naim, Erika Bauza Nowotny, Janet Moradian-Oldak)**

# Supplementary Information


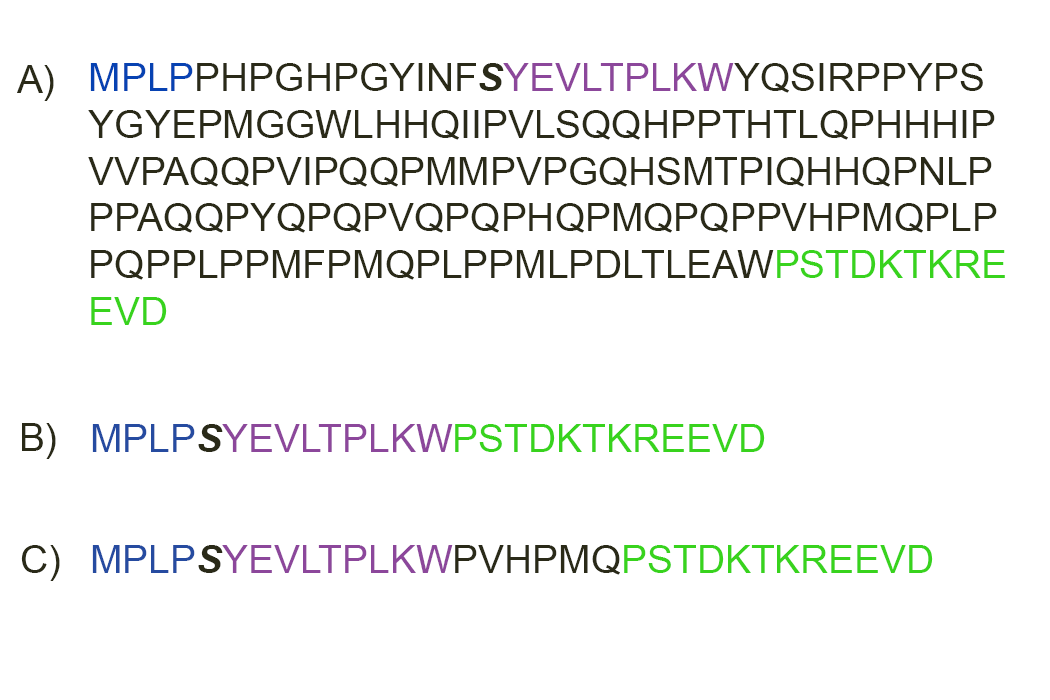


**Supplementary Fig. 1.** Amino acid sequences of (**A**) full-length human amelogenin (H174) (**B**) P26 peptide and (**C**) P32 peptide. P26 and P32 contain sequences from functional domains of rH174 including N-terminus (blue), hydrophobic core (purple), and C-terminus (green) of H174. All peptide sequences include the only post-translational modification (phosphorylation) on serine (pS^16^).

**
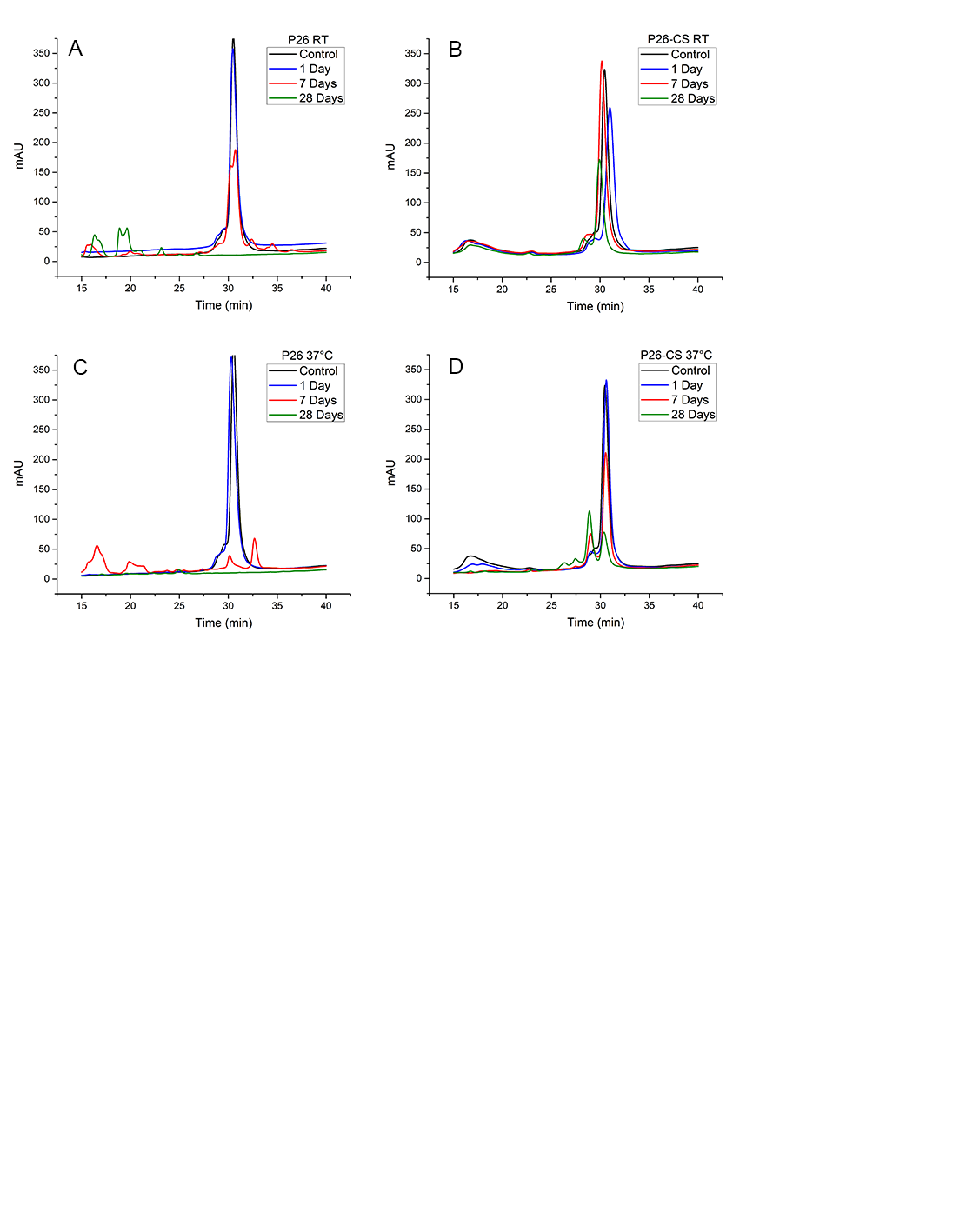
**

**Supplementary Fig 2.** Representative HPLC profiles of the amelogenin peptide peak at

0 (control), 1, 7, and 28 days. The amelogenin peptide peak (min. 30-31) area remained largely unchanged for P26 and P26-CS at RT (**A,B**, respectively) and 37°C (**C,D**, respectively), indicating substantial stability of the peptides after 1 day. P26 exhibited a remarkable reduction in peak area after 7 days at RT (a) and later disappearance of the peak along with appearance of smaller peaks after 28 days (**A,C**). In contrast, the amelogenin peptide peak in P26-CS was present at 28 days at RT (**B**) and 37°C (**D**) denoting greater stability than its P26 counterpart. Some samples exhibited experimental error (<10%) represented by slight variations in peak elution times.

**
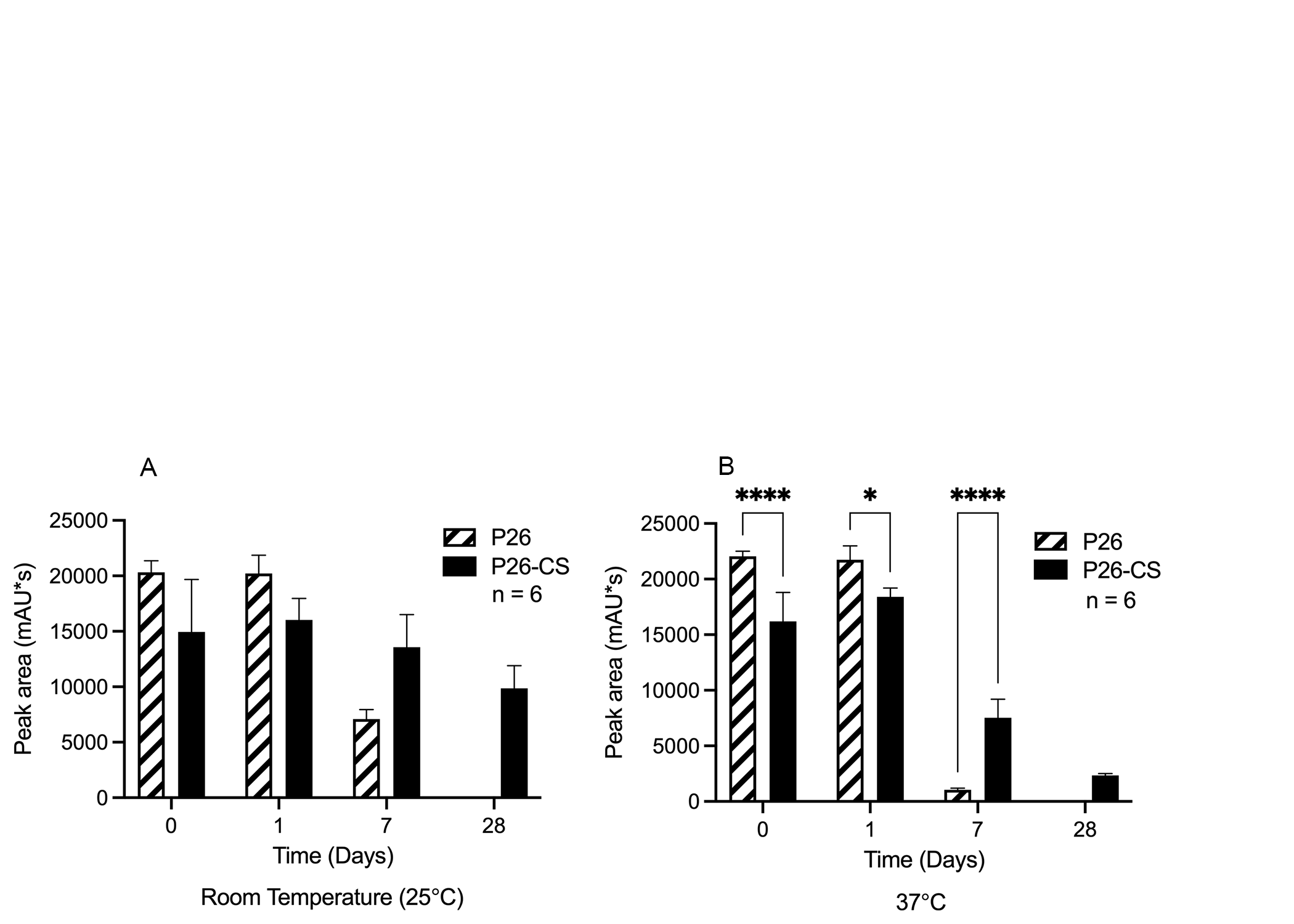
**

**Supplementary Fig. 3:** Amelogenin peptide peak area of P26 and P26-CS. Samples were collected and analyzed via HPLC after incubation at RT (**A**) and 37°C (**B**) for 1, 7, and 28 days. A significant effect was observed between treatment (P26 or P26-CS) and time (*p* < 0.0001) by two-way ANOVA. Pairwise Bonferroni adjusted comparisons showed that the mean peak area was significantly different between P26 and P26-CS after 0 days (*p* < 0.0001), 1 day (*p* = 0.0165), and 7 days (*p* < 0.0001) at 37 °C. Average values of triplicates are shown with error bars depicting standard deviation. *≤ 0.05; ****≤ 0.0001.
